# The conundrum of using hyperoxia in COVID-19 treatment strategies: may intermittent therapeutic hyperoxia play a helpful role in the expression of the surface receptors ACE2 and Furin in lung tissue via triggering of HIF-1α?

**DOI:** 10.1186/s40635-020-00323-1

**Published:** 2020-09-15

**Authors:** Andreas Koch, Wataru Kähler, Sebastian Klapa, Bente Grams, Pieter- Jan A. M. van Ooij

**Affiliations:** 1 Joint Section of Maritime Medicine, Naval Institute of Maritime Medicine and Christian-Albrechts-University Kiel, Kiel, Germany; 2 Diving Medical Center, Royal Netherlands Navy, Den Helder, and Respiratory Department of the Academic Medical Center, Amsterdam, the Netherlands

## Introduction

In the current pandemic of severe acute respiratory syndrome corona virus 2 (SARS-CoV-2), the therapeutic administration of oxygen is a common procedure in order to mitigate patient’s hypoxia in the course of severe corona virus disease 2019 (COVID-19) pneumonia. However, additional oxygen causes a variety of well-known side-effects, impacting a number of systems regulating cardiovascular and respiratory homeostasis as well as reactive oxygen species (ROS)-production via oxidative stress.

In this article, we want to focus on intermittent changes in lung and tissue oxygenation, as changes in local pO_2_ may be able to trigger one of the key effectors of cellular oxygen-sensing, hypoxia-inducible factor-1α (HIF-1α) and, in downstream, the expression of angiotensin-converting enzyme-2 (ACE2) and Furin.

Recently, evidence has been found that the ACE2-receptor in lung tissue, in combination with Furin, is essential for the development of COVID-19 [1–3].

Besides, there is also new information, that in contrast a severe course of lung complication of COVID-19 is correlated with reduced ACE2 on the cell surface due to shedding of the ACE2-receptor from the cell surface [4] and thus, with an insufficient angiotensin-(2) (AT2)-conversion to angiotensin-(1-7) (AT1-7).

This finding, that reduced ACE2 on lung cell surfaces is correlated with lung damage due to an uncontrolled renin-angiotensin system (RAS) cascade, is supported by recent data about effects of long-lasting hyperoxia on pulmonary tissue [5–10].

Thus, in the course of COVID-19, ACE2 plays a complex role: high levels of ACE2-receptors on the cell surface in combination with Furin may facilitate SARS-Cov2-intrusion during the very early phase of infection, but in case of severe COVID-19 with pulmonary complications finally low levels of ACE2 can finally worsen the situation due to insufficient AT2-conversion.

In both situations, in the beginning and in the pulmonary complication phase, manipulation of inspiratory pO_2_ may be a way to influence the levels of ACE2 and Furin with beneficial effects on the further course of the disease.

### Role of angiotensin-converting enzyme-2 in the course of COVID-19

Like in many other tissues, lung cells also have a local RAS [11], which influences the pathogenesis of lung injury via cellular effects, like changes in vascular permeability, vascular tone, fibroblast activity, or alveolar epithelial cell apoptosis [12, 13]. ACE2 plays a pivotal role in the RAS cascade starting with angiotensinogen and ending with AT1-7 [11]. The latter enzyme has a downregulating effect on this RAS cascade and thus limits inflammation and fibrosis [11, 14]. However, a decrease in ACE2 activity, like in longer-lasting hyperoxia, diminishes the conversion of AT2 into AT1-7, thus leading to an increment of AT2 [7]. The increased presence of AT2 initiates, among others, the development of fibrosis in the lung and in addition, might significantly contribute to an uncontrolled inflammation, e.g., severe increases in interleukin 6 (IL-6) [15].

As described above, a similar decrease in ACE2 has also been seen in COVID-19 with severe pulmonary complications, which might initiate comparable negative consequences, that arise from insufficient AT2-inactivation [4].

### Role of Furin in the course of COVID-19

Furin, also known as paired basic amino acid cleaving enzyme (PACE) or proprotein convertase subtilisin/kexin type-3 (PCSK3), is identified as the prototype of the currently known mammalian subtilisin/kexin-like proprotein convertases and is involved in the activation of precursors of growth factors, viral glycoproteins, and adhesion receptors via endoproteolytic cleavage at basic amino acid residues [16, 17]. In detail, bioinformatic studies identified more than 100 Furin cleavage sites in mammalian proteins, e.g., cytokines, hormones, receptors, and albumin [18]. On the other hand, most viral envelope glycoproteins need to be proteolytically cleaved for mediating the viral entry into the host cells, such as herpesvirus, retrovirus, or corona virus [17]. Indeed, in COVID-19, the cleavage function of Furin facilitates the penetration of SARS-CoV-2 into the cell after binding to the ACE2-receptor [2, 19]. The localization of Furin involves intracellular compartments as well as the cell surface and may explain its ability to process an expanded variety of substrates. In animal models, for instance, it is essential in the astrocyte neuroprotection mechanisms during hypoxia in acute cerebral ischemia [20].

### COVID-19 and oxygen therapy

In the current pandemic situation of SARS-CoV2, there are, among all the other numerous therapeutic attempts, also approaches using (hyperbaric) hyperoxia as an additional option in the treatment of patients suffering COVID-19. Currently, there are seven clinical trials in preparation using hyperbaric oxygenation therapy (HBOT) as a therapeutic (adjuvant) option in COVID-19 patients with respiratory distress (NCT04343183; NCT0433208; NCT04344431; NCT04358926; NCT04386265; NCT04409886; NCT04327505).

However, nearly nothing is even known about the effect of therapeutic administered oxygen on the expression of ACE2 and Furin in respiratory tissues.

Besides, there is a body of evidence from diving and hyperbaric medicine, intensive care, and neonatology that hyperoxia is able to harm the lung [21].

In diving as well as in hyperbaric oxygen therapy (HBOT), oxygen is delivered to the body with increased partial pressures, ranging from slightly supranormal in SCUBA-diving to up to more than the 15-fold in HBOT [22].

In diving medicine and HBOT, the hyperoxia-dependent risk of lung damage is calculated by the UPTD-formula (units of pulmonary toxicity dose), and similar risk assessments are used in anesthesiology and intensive care medicine [23, 24].

Hyperoxia-induced lung reactions may range from early signs of inflammation up to severe impairment of lung function and are discussed to be one of the major contributors to the development of the acquired respiratory distress syndrome (ARDS) as a complication of long-time ventilation in intensive care [21, 25].

Furthermore, in neonatology, hyperoxia is known to have the capability of inducing severe lung reactions, which, in the end, can result in lung fibrosis [8, 10, 26].

In addition, experimental data show that longer-lasting administration of oxygen to fetal lung cells results in a downregulation of ACE2 and is related to the development of lung fibrosis [8–10].

In this context of hyperoxia-inducible lung damage and the certain role of ACE2 in its pathophysiology, there is a need to closer focus on the role of HIF-1α, since it plays a key role in cellular oxygen sensing and is one of the main factors regulating the expression of ACE2 and Furin.

### Role of HIF-1α in cellular oxygen sensing

The substantial mechanisms responsible for hyperoxia-induced lung damage are still under investigation with a certain role of oxidative stress and a number of further mechanisms, which include pathways of hypoxia-sensing and reaction. More importantly, there is information that the transcription factor HIF-1⍺ and its induced factors ACE2 and Furin may thereby play a significant role [7].

HIF-1⍺, whose expression is known to be dependent from cellular oxygen supply, has been identified to be one factor regulating the mRNA-expression for the synthesis of ACE2 and Furin [27–29].

Hypoxia is the natural trigger for upregulation of HIF-1⍺ [30, 31]; however, there is evidence that this effect attenuates over time [30]. Vice versa, in longer-lasting hyperoxia, HIF-1⍺ becomes downregulated [32–34].

Noteworthy, there is some evidence that *intermittent hyperoxia*, i.e., changes between phases with elevated pO_2_ and prior conditions (e.g., in HBOT), can also act as a trigger to increase HIF-1⍺-level [35]. This phenomenon, formerly entitled as “normobaric oxygen paradox,” is discussed to be due to *relative hypoxia* after short phases of elevated oxygen tension [36].

As one central sensing mechanism, increased intramitochondrial ROS-production due to hypoxia as well as intermittent hyperoxia may initiate stabilization of active HIF-1⍺ [37–39], which induces increased ACE2- and Furin-expression [27–29].

## Hypothesis

As HIF-1⍺ and in consequence HIF-1 seem to be central factors for the expression of ACE2 and Furin, and as the stabilization of active HIF-1⍺ is oxygen-dependent, the therapeutic administration of oxygen might have a significant influence on ACE2- and Furin-expression.

Two different consequences can be drawn from the above-mentioned interactions between changes in pO_2_ and the expression of HIF-1α:

First, a longer-lasting administration of additional oxygen may induce a reduction in HIF-1α and in consequence in ACE2 and Furin. A reduced number of binding sites for the SARS-CoV-2 virus may be beneficial in the very early phase of the infection with respect to virus-intrusion.

However, it is questionable, whether the administration of hyperoxia is practicable in the very early infection phase, even in risk patients.

Second, since both manifest hypoxia itself as well as administration of intermittent hyperoxia with consecutive relative hypoxia occurring after completion, seem to be able to induce an increased HIF-1α-expression, this mechanism may be a chance to increase critically reduced levels of ACE2 and Furin in case of severe pulmonary complications. However, in severe pneumonia, the administration of supplemental oxygen is often essential in order to avoid tissue hypoxia in the patient, and thus, a triggering of HIF-1α-expression via a withholding of oxygen seems not to be feasible.

On the other hand, since intermittent phases of hyperoxia with consecutive-only relative hypoxia may also result in a HIF-1α-induction similar to real hypoxia, the administration of phases with intermittent hyperoxia on the ICU seems to be a chance to increase the critically reduced ACE2-receptor levels in severe COVID-19 pneumonia. This might help to re-establish adequate AT2-conversion into AT(1-7) and thus, to preserve a functional RAS-cascade. Whether a parallel triggered increase in Furin may have additional positive effects is unknown and needs to be elucidated.

Typical hyperbaric oxygenation therapy (HBOT; 2.0–2.5ata) sessions with their changes between 100% oxygen and “air breaks” (short phases of ambient air for pulmonary protection, depending on the used schedule) under pressure during therapy and “only” normobaric conditions after completion may induce a number of relative hypoxic phases. Thus, besides overall enhanced tissue oxygenation, these relative hypoxic phases may induce the discussed increase in HIF-1α-expression over time.

Up to now, this control circuit has scarcely been investigated in relation with the possible clinical consequences in COVID-19-therapy. Yet, there might be a chance that the administration of intermittent short phases of hyperoxia may be helpful to reduce some complications of COVID-19 pneumonia when the development of the clinical picture makes it necessary to take critically reduced ACE2-levels into account.

Further basic research in human cells and tissues is needed to precisely elucidate the underlying mechanisms and its usability as an additional therapy for COVID-19 in both, the very early infection and the severe COVID-19 pneumonia.

## Data Availability

Not applicable.

## Abbreviations

ACE2: Angiotensin-converting enzyme-2
ARDS: Acquired respiratory distress syndrome
AT1-7: Angiotensin-(1-7)
AT2: Angiotensin-(2)
COVID-19: Corona virus disease 2019
HBOT: Hyperbaric oxygen therapy
HIF-1: Hypoxia-inducible factor-1
HIF-1α: Hypoxia-inducible factor-1α
mRNA: Messenger-ribonucleic acid
PACE: Paired basic amino acid cleaving enzyme
PCSK3: Proprotein convertase subtilisin/kexin type-3
RAS: Renin-angiotensin system
ROS: Reactive oxygen species
SARS-CoV-2: Severe acute respiratory syndrome corona virus 2
SCUBA: Self-containing underwater breathing apparatus
UPTD: Units of pulmonary toxicity dose
